# Revisiting global health education: The engagement of medical students

**DOI:** 10.7189/jogh.13.03059

**Published:** 2023-11-03

**Authors:** Chaoyu Lei, Hui Zhou, Mingyu Qu, Haoxuan Cheng, Zecheng Tao, Xuefei Song, Huifang Zhou, Lufa Zhang

**Affiliations:** 1 Department of Ophthalmology, Ninth People’s Hospital, Shanghai Jiao Tong University School of Medicine, Shanghai, China; 2 Shanghai Key Laboratory of Orbital Diseases and Ocular Oncology, Shanghai, China; 3 School of International and Public Affairs, Shanghai Jiao Tong University, Shanghai, China; 4 Institute for Urban Governance, Shanghai Jiao Tong University, Shanghai, China; 5 Institute of Healthy Yangtze River Delta, Shanghai Jiao Tong University, Shanghai, China

**Figure:**
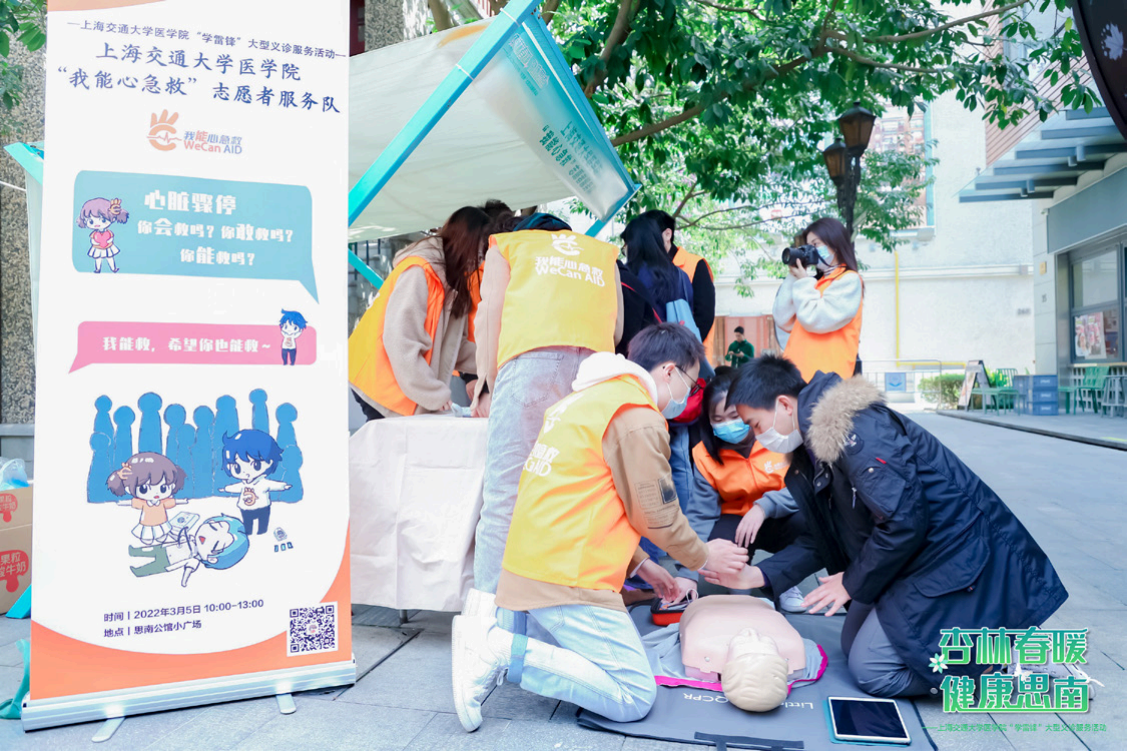
Photo: Medical students delivering first-aid health information to the public. Used with the permission of the photo author/copyright holder: Dalen Raymond.

In the midst of several global public health crises this century, the lack of health literacy has greatly hindered the implementation of public health policies. Prior research has demonstrated positive correlations between health literacy, health engagement, and health [1]. Effective health information dissemination can proactively guide the public to practice health behaviours and ultimately contribute to positive health outcomes [2,3]. Besides, in light of the widespread adoption of online technology and the escalating public health risks, the demand for quality health education is increasing. The current scope of health education, however, falls short of meeting these demands. According to our questionnaire study in 20 provinces in China in 2022, 93.85% of participants acknowledged the necessity of health education related to eye health, yet only 30.00% have received effective eye-related health education. Therefore, the inadequacy of public health education, particularly in low- and middle-income countries, remains glaring.

## MEDICAL STUDENTS ARE POTENTIAL PROVIDERS OF PUBLIC HEALTH EDUCATION

The supply-demand disequilibrium of public health education can be attributed to the fact that the main actor responsible for health education is still unclear. To clarify the primary actors responsible for health education, it is noted that health education needs to be scientific and professional but also effective in reaching the public. The media and medical practitioners were traditionally perceived to bear the main responsibility for health education. However, the media lacks the expertise in health science [4]. In addition, the media’s misleading discourse on scientific information can lead to public misunderstanding and mistrust [4]. Medical practitioners possess professional knowledge, but they usually underestimate the significance of health education and lack institutional incentives and training in communication skills to conduct health education [5]. It is also challenging for medical practitioners to devote their time to health education in underprivileged regions [6].

In contrast to these two groups, medical students, who are often overlooked as potential providers of public health education, have the willingness, ability, and time to engage in health education. First, medical students have the motivation to solve real-life medical needs and have laid a potentially solid foundation in health science knowledge. Their involvement in health education can enhance their sense of professional responsibility and empower their future careers. Second, medical students are in a transition phase from the general public to health professionals [4]. They have the ability to translate specialised medical knowledge into plain language and communicate this knowledge to the public. Third, medical students do not yet have heavy workloads in health care service delivery and can spend their time on health education. It provides a valuable opportunity for medical students to meet patients, familiarise themselves with medical practices, and develop communication skills between physicians and patients.

## HEALTH POLICY RECOMMENDATIONS

The current medical training system has not fully acknowledged the important role of medical students in disseminating health education to a broader population. We propose to recognise that medical students can and should actively join the cause of promoting health literacy. This initiative still requires broad and robust support from health systems, education systems, and the international community, which leads to the following global health policy recommendations.

Make the government responsible for guiding health education centred on medical students and enhance multi-sectoral collaboration. This includes improving the health education policy system, mobilising medical schools, professors, and practitioners in guiding medical students, supporting social organisations to launch health education initiatives, and encouraging medical students to participate and lead related activities progressively.

Make the best use of universities’ resources to enhance interdisciplinary collaboration among medical students. Universities should construct a multidisciplinary health education platform covering teaching, practice and assessment, where students majoring in medicine, social science, and humanities can voluntarily put forward population-based and context-related health education strategies. In addition, health education should be incorporated into extra-curricular activities or credited courses for medical students. Associations and campaigns can be set up to enhance medical students’ incentives for health education.

Facilitate medical students’ participation in health education for broader equality in health literacy. Inequality in health literacy is one of the components of global health inequalities that impede public health decision-making [7]. It is encouraged to integrate information technology into low-cost, high-impact mass media campaigns to extend multiple pathways of dissemination, such as health education web pages and animated videos. Meanwhile, de facto accessibility and affordability of health information need to be underlined to prevent widening inequalities.

## CONCLUSION

The accessibility and availability of health knowledge are underestimated, yet they constitute the cornerstone for a resilient global health system. Therefore, this paper underscores the pivotal role of medical students in advancing health literacy and providing them with a learning environment to develop relevant competencies. Health education provided by medical students can thus effectively bridge the knowledge gap, prevent misconceptions about diseases, and improve the public’s capacity for informed health decision-making.
